# Anti-cancer natural products isolated from chinese medicinal herbs

**DOI:** 10.1186/1749-8546-6-27

**Published:** 2011-07-22

**Authors:** Wen Tan, Jinjian Lu, Mingqing Huang, Yingbo Li, Meiwan Chen, Guosheng Wu, Jian Gong, Zhangfeng Zhong, Zengtao Xu, Yuanye Dang, Jiajie Guo, Xiuping Chen, Yitao Wang

**Affiliations:** 1 State Key Laboratory of Quality Research in Chinese Medicine, University of Macau, Av. Padre Toma's Pereira S.J., Taipa, Macao SAR, China; 2 Institute of Chinese Medical Sciences, University of Macau, Av. Padre Toma's Pereira S.J., Taipa, Macao SAR, China; 3 College of Life Sciences, Zhejiang Chinese Medical University, 548 Binwen Rd., Binjiang Dist., Hangzhou 310053, Zhejiang, China; 4 College of Pharmacy, Fujian University of Traditional Chinese Medicine, No.1 Huatuo Rd., Shangjie University Town, Fuzhou 350108, Fujian, China

## Abstract

In recent years, a number of natural products isolated from Chinese herbs have been found to inhibit proliferation, induce apoptosis, suppress angiogenesis, retard metastasis and enhance chemotherapy, exhibiting anti-cancer potential both *in vitro *and *in vivo*. This article summarizes recent advances in *in vitro *and *in vivo *research on the anti-cancer effects and related mechanisms of some promising natural products. These natural products are also reviewed for their therapeutic potentials, including flavonoids (gambogic acid, curcumin, wogonin and silibinin), alkaloids (berberine), terpenes (artemisinin, β-elemene, oridonin, triptolide, and ursolic acid), quinones (shikonin and emodin) and saponins (ginsenoside Rg_3_), which are isolated from Chinese medicinal herbs. In particular, the discovery of the new use of artemisinin derivatives as excellent anti-cancer drugs is also reviewed.

## Background

Surgery, chemotherapy and radiotherapy are the main conventional cancer treatment often supplemented by other complementary and alternative therapies in China [1]. While chemotherapy is one of the most extensively studied methods in anti-cancer therapies, its efficacy and safety remain a primary concern as toxicity and other side effects of chemotherapy are severe. Moreover, multi-drug resistant cancer is even a bigger challenge. Medicinal herbs are main sources of new drugs. Newman *et al. *reported that more than half of the new chemicals approved between 1982 and 2002 were derived directly or indirectly from natural products [2]. Some active compounds have been isolated from Chinese medicinal herbs and tested for anti-cancer effects. For example, β-elemene, a compound isolated from *Curcuma wenyujin *Y. H. Chen *et *C. Ling (Wenyujin), is used as an anti-cancer drug in China. For this study, we searched three databases, namely PubMed, Scopus and Web of Science, using keywords "cancer", "tumor", "neoplastic" and "Chinese herbs" or "Chinese medicine". Publications including research and review papers covered in this review were dated between 1987 and 2011, the majority of which were published between 2007 and 2011. Chinese herb-derived ingredients, including flavonoids, alkaloids, terpenes, quinones and saponins, were found.

### Gambogic acid (GA)

**Figure 1:**
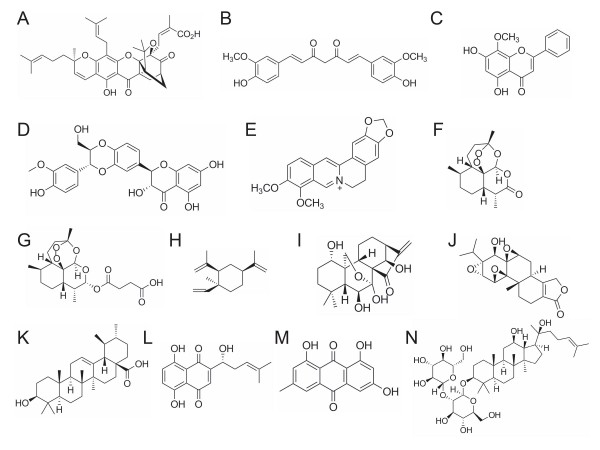
**Chemical structures of the compounds**. (A) gambogic acid; (B) curcumin; (C) wogonin; (D) silibinin; (E) berberine; (F) artemisinin; (G) artesunate; (H) β-elemene; (I) oridonin; (J) triptolide; (K) ursolic acid; (L) shikonin; (M) emodin; (N) ginsenoside Rg_3_.

GA (Figure 1A) is the principal active ingredient of gamboges which is the resin from various *Garcinia *species including *Garcinia hanburyi *Hook.f. (*Tenghuang*) [3]. GA has various biological effects, such as anti-inflammatory, analgesic and anti-pyretic [3] as well as anti-cancer activities [4,5]. *In vitro *and *in vivo *studies have demonstrated its potential as an excellent cytotoxicity against a variety of malignant tumors, including glioblastoma, as well as cancers of the breast, lung and liver. GA is currently investigated in clinical trials in China [6-8].

GA induces apoptosis in various cancer cell types and the action mechanisms of GA remain unclear. Transferrin receptor (TfR) significantly over-expressed in a variety of cancers cells may be the primary target of GA [4]. The binding of GA to TfR in a manner independent of the transferrin binding site, leading to the rapid apoptosis of tumor cells [4]. Proteomics analysis suggests that stathmin may be another molecular target of GA [9]. The importance of the role of p53 in GA-induced apoptosis remains controversial [5,10]. Furthermore, GA antagonizes the anti-apoptotic B-cell lymphoma 2 (Bcl-2) family of proteins and inhibits all six human Bcl-2 proteins to various extents, most potently inhibiting myeloid cell leukemia sequence 1 (Mcl-1) and Bcl-B, as evidenced by a half maximal inhibitory concentration (IC_50_) lower than 1 μM [11]. Moreover, GA also influences other anti-cancer targets, such as nuclear factor-kappa B (NF-κB) [12] and topoisomerase IIα [13].

GA causes a dose-dependent suppression of cell invasion and inhibits lung metastases of MDA-MB-435 cells *in vivo *through protein kinase C (PKC)-mediated matrix metalloproteinase-2 (MMP-2) and matrix metallopeptidase-9 (MMP-9) inhibition [8]. GA also exhibits significant anti-metastatic activities on B16-F10 melanoma cancer cells partially through the inhibition of the cell surface expression of integrin α4 in *C57BL/6 *mice [14].

Notably, the combination of GA with other compounds enhances their anti-cancer activities [15-17]. For example, He *et al. *[15] reports that proliferative inhibition and apoptosis induction are much more visibly increased when Tca8113 cells are treated with combined GA and celastrol, indicating that the combination of GA and celastrol can be a promising modality for treating oral squamous cell carcinoma. Another study showed that GA in combined use with 5-fluorouracil (5-FU) induced considerably higher apoptosis rates in BGC-823 human gastric cells and inhibited tumor growth in human xenografts [16]. Furthermore, low concentrations of GA were found to cause a dramatic increase in docetaxel-induced cytotoxicity in docetaxel-resistant BGC-823/Doc cells [17]. Magnetic nanoparticles of Fe_3_O_4 _(MNPs-Fe_3_O_4_) were reported to enhance GA-induced cytotoxicity and apoptosis in K562 human leukemia cells [18].

### Curcumin

Curcumin (Figure 1B) is the main active flavonoid derived from the rhizome of *Curcuma longa *(*Jianghuang*), with its dry herb weight consisting of up to 3.08% curcumin [19]. Curcumin has been used to treat cardiovascular disease, inflammation and arthritis [20]. Epidemiological studies have found that incidence of several cancers is low in India where curcumin is widely consumed, suggesting that curcumin intake plays a role in cancer prevention [21]. Other studies have also indicated that curcumin inhibits cell proliferation and survival in breast cancer, colon cancer, prostate cancer, gastric cancer, leukemia, lymphoma and melanoma [20]. Curcumin induces cell apoptosis through complex intrinsic and extrinsic pathways. Curcumin binds to more than 30 different protein targets, including transcript factors (NF-κB and activator protein-1), growth factor receptors [epidermal growth factor receptor (EGFR), human epidermal growth factor receptor 2 (HER2)], kinases [mitogen-activated protein kinase (MAPK), PKC and protein kinase A (PKA)], inflammatory cytokines [tumor necrosis factor (TNF) and interleukins], cell cycle-related proteins (p53 and p21), matrix metalloproteinases (MMPs) and urokinase plasminogen activators (u-PA) [20,22,23]. Daily oral administration of curcumin suppresses metastasis in breast, colon, lung and medulloblastoma cancers. The suppression involves the regulation of metastatic proteins, such as vascular endothelial growth factor (VEGF), MMP-2, MMP-9 and intercellular adhesion molecules [24,25].

Curcumin induces non-apoptotic cell death, such as autophagic cell death, which involves the degradation of the cell's own components through lysosomal machinery [23]. *In vitro *and *in vivo *studies have demonstrated that curcumin induces autophagic cell death, as evidenced by the immunoreactivity of microtubule-associated protein light chain 3 (LC3) in myeloid leukemia cells. The action mechanism is attributed to the inhibition of the Akt/mammalian target of rapamycin/p70 ribosomal protein S6 kinase pathway and activation of extracellular signal-regulated kinase 1/2 by curcumin in malignant glioma cells [26,27]. In addition, autophagic inhibitor bafilomycin A1 suppresses curcumin-induced cell death [28]. Another type of non-apoptotic cell death induced by curcumin is paraptosis which is observed in malignant breast cancer cells but not in normal breast cells. Curcumin induces paraptotic events (*eg *the promotion of vacuolation accompanied with mitochondrial and/or endoplasmic reticular swelling and fusion) and decreases the level of paraptotic inhibitor protein AIP-1/Alix [29]. These paraptotic events are attributed to superoxide anion and proteasomal dysfunction [29].

Curcumin reduces toxicity induced by anti-cancer agents [30], sensitizes chemo-resistant cancer cells and demonstrates synergic effects with different chemotherapeutic agents such as doxorubicin, 5-FU, paclitaxel, vincristine, melphalan, butyrate, cisplatin, celecoxib, vinorelbine, gemcitabine, oxaliplatin, etoposide, sulfinosine, thalidomide, suberoylanilide hydroxamic acid, dasatinib and bortezomib [30]. Prior administration of curcumin reduces the DNA damage and oxidative stress induced by cyclophosphamide (CXC) [31], improves uroprotective efficacy in the CXC hemorrhagic cystitis model [32] and suppresses early lung damage in CXC-treated rats [33]. Curcumin alleviates the side effects of mitomycin C, as evidenced by decreased lipid peroxidation and DNA damage [34]. Furthermore, curcumin reduces weight loss and improves kidney function and bone marrow suppression in animal studies [35]. When combined with oxaliplatin, curcumin decreases the proliferative capacity of oxaliplatin-resistant cell lines and enhances the cytotoxicity of oxaliplatin in an *in vitro *oxaliplatin-resistant model [36]. Additionally, curcumin protects healthy cells against radiation and sensitizes tumor cells to radiation therapy [37,38].

Clinical trials have been or are currently being conducted to evaluate the tolerance, safety, pharmacokinetics and efficiency of curcumin as well as its combination therapy with current anti-cancer drugs [39]. A phase I clinical trial found no dose-limiting toxicity in patients treated with an oral-dose of up to 8g/day of curcumin. The recommendation is seven consecutive doses (6g/day) of curcumin every three weeks in combination with a standard dose of docetaxel [40]. Improvements in biological and clinical responses were observed in most treated patients [40]. A phase II trial of gemcitabine-resistant pancreatic cancer found chemotherapeutic drugs in combined use with curcumin to be sufficiently safe, feasible and efficient. While the bioavailability of curcumin is relatively poor, two out of 21 patients in the phase II trial showed clinical biological responses; one patient exhibited marked tumor regression coupled with a significant increase in serum cytokine levels [41,42].

### Wogonin

Wogonin (Figure 1C) is one of the flavonoids isolated from *Scutellaria baicalensis *Georgi (*Huangqin*), with its dry herb weight consisting of up to 0.39 mg/100 mg of wogonin [43]. Wogonin has been widely used in the treatment of various inflammatory diseases owing to its inhibition of nitric oxide (NO), prostaglandin E_2 _and pro-inflammatory cytokines production, as well as its reduction of cyclooxygenase-2 (COX-2). *In vitro *studies [44-48] have shown wogonin to possess cytostatic and cytotoxic activities against several human tumor cell lines.

Wogonin induces apoptosis through the mediation of Ca^2+ ^and/or inhibition of NF-κB, shifting O_2_^- ^to H_2_O_2 _to some extent; H_2_O_2_, in turn, serves as a signaling molecule that activates phospholipase Cγ. Ca^2+ ^efflux from the endoplasmic reticulum is then regulated, leading to the activation of Bcl-2-associated agonist of cell death [44]. Wogonin may also directly activate the mitochondrial Ca^2+ ^channel uniporter and enhance Ca^2+ ^uptake, resulting in Ca^2+ ^overload and mitochondrial damage [44]. Furthermore, wogonin induces cell type-dependent cell cycle inhibitions in cancer cells, such as those observed in human cervical carcinoma HeLa cells at the G_1 _phase [48] and in THP-1 cells at the G_2_/M phase [46] respectively. Unlike the inhibitory effect of baicalein and baicalin on normal human fetal lung diploid TIG-1 cells [46], wogonin imposes minor or almost no toxicity on normal peripheral T cells [44], TIG-1 cells [46] and human prostate epithelial cells [47]. This selective inhibition of wogonin is due to a high expression of L-type voltage dependent Ca^2+ ^channels in cancer cells [44]. In addition, wogonin suppresses VEGF-stimulated migration and tube formation in HUVEC by inhibiting VEGF receptor 2 (VEGFR2) instead of VEGFR1 phosphorylation [49].

The synergistic effect of wogonin on chemotherapy drugs, such as etoposide, has also been investigated. Wogonin significantly improves etoposide-induced apoptosis in cancer cells in a similar capacity as the typical P-glycoprotein (P-gp) inhibitors verapamil and cyclosporine A [50-52]. However, other P-gp substrates, such as doxorubicin and vinblastine, do not show any synergistic effect [52]. Similar effect was also found when combination treatment with 5-FU in human gastric MGC-803 cells and in MGC-803 transplanted nude mice [53]. The underlying mechanisms might be due to its pro-apoptotic effect and inhibition of NF-κB nuclear translocation activity [53].

Anti-inflammatory and anti-viral activities of wogonin may also contribute to tumor prevention [54]. Wogonin is a good anti-cancer candidate due to its broad toxicities to various types of tumor cell lines and the low toxicities to normal tissues, as well as the synergistic effects.

### Silibinin

Silibinin (Figure 1D), a mixture of flavonoids derived from *Silybum marianum *(*Shuifeiji*), is therapeutically used for the treatment of hepatic diseases in China, Germany and Japan. Silibinin has effects on many cancers, such as prostate, colon, bladder and lung cancers [55,56], particularly the migration, invasion and metastasis of cancer cells [57]. In a transgenic adenocarcinoma of the mouse prostate (TRAMP) mouse model, silibinin inhibits tumor growth, progression, local invasion and distant metastasis [56]. Silibinin induces both death receptor-mediated and mitochondrial-mediated apoptosis in human breast cancer MCF-7 cells [58]. Silibinin also reduces hepatocellular carcinoma xenograft growth through the inhibition of cell proliferation, cell cycle progression, as well as phosphatase and tensin homolog/P-Akt and extracellular signal-regulated kinase (ERK) signaling. These effects induce apoptosis and increase histone acetylation and superoxide dismutase-1 (SOD-1) expression on human hepatocellular carcinoma xenografts [59]. Not only does silibinin inhibit primary prostatic tumor progress but also protects against angiogenesis and late-stage metastasis. Therefore, silibinin may have a potential for improving survival and reducing morbidity in prostate cancer patients [60].

Silibinin exerts anti-cancer activity mainly by blocking cell cycle progression and induces G1 cell cycle arrest in a dose- and time-dependent manner in large cell carcinoma H1299 and H460 cells and bronchioalveolar carcinoma H322 cells [61]. Silibinin modulates the protein levels of cyclin-dependent kinases (CDKs; 4, 6 and 2), cyclins (D1, D3 and E), and CDK inhibitors (p18/INK4C, p21/Waf1 and p27/Kip1) in a differential manner in the above-mentioned cell lines [61]. Silibinin also regulates multiple cellular proliferative pathways in cancer cells, including receptor tyrosine kinases (RTKs), androgen receptors, signal transducers and activators of transcription (STATs), NF-κB [62]. Moreover, silibinin inhibits the constitutive activation of STAT3 and causes caspase activation and apoptotic cell death in human prostate carcinoma DU145 cells [63].

The combined use of silibinin with 1,25-dihydroxyvitamin D3 promotes the expression of both differentiation-promoting and -inhibiting genes in acute myelogenous leukemia cells and the latter can be neutralized by a highly specific pharmacological inhibitor, suggesting the therapeutic potential of silibinin [64].

### Berberine

Berberine (Figure 1E) is an isoquinoline alkaloid isolated from *Coptidis Rhizoma *(*Huanglian*), which is a Chinese medicinal herb for heat dissipation and detoxification, with its dry herb weight consisting of up to 7.1 mg/100 mg of berberine [65]. Berberine has diverse pharmacological activities [66-70] and is especially used as an antibacterial and anti-inflammatory gastrointestinal remedy in China [71]. Berberine has anti-proliferative effects on cancer cells has been documented [72-78]. Multiple targets of berberine have been identified, including mitochondria, DNA or RNA, DNA topoisomerases, estrogen receptors, MMPs, p53 and NF-κB [74,79-82]. Berberine exerts cytotoxicity and inhibits telomerase and topoisomerase in cancer cells by specifically binding to oligonucleotides or polymorphic nucleic acid and by stabilizing DNA triplexes or G-quadruplexes [81,83,84]; the electrostatic interactions may be quantified in terms of the Hill model of cooperative interactions [85].

Cell cycle regulation is a common target mechanism in anti-cancer therapies. A low-dose (12.5-50 μM) berberine treatment induces G1 phase arrest whereas doses higher than 50 μM induce G2 phase arrest in mouse melanoma K1735-M2 and human melanoma WM793 cells [86]. Moreover, 50 μM berberine decreases cyclin B1 levels and induces cycle arrest at the G1 phase in human lung cancer H1299 and A549 cell lines [75]. Even in anoikis-resistant human breast cancer MDA-MB-231 and MCF-7 cells, 10 or 20 μM doses of berberine is superior to 5 or 10 nM of doxorubicine respectively by inducing cell cycle arrest at the G0/G1 phase [87].

In human breast cancer MCF-7 cells, berberine induces apoptosis through a mitochondrial dependent pathway by increasing the Bcl-2-associated × protein (Bax)/Bcl-2 protein ratio, activating caspases and inducing poly (ADP-ribose) polymerase (PARP) cleavage [76]. These apoptotic processes also occur in human tongue squamous carcinoma cancer-4 and human glioblastoma T98G cells [73,88]. Accumulation of berberine on mitochondrial membranes alters the binding between adenine nucleotide translocator and bongkrekic acid, thereby inducing depolarization and fragmentation which may contribute to mitochondrial respiration inhibition and mitochondrial dysfunction [89]. In the p53-expressing human neuroblastoma SK-N-SH and p53-deficient SK-N-MC cells, the role of p53 in berberine's anti-neoplastic function is highlighted by the cytotoxic effects and apoptotic gene expression accompanied by caspase-3 activation [72].

In addition to apoptotic alteration induced by berberine, recent findings are about anti-cancer mechanisms that have a higher propensity to cause autophagy. Berberine induces autophagic cell death in human hepatocellular liver carcinoma cell lines (HepG2) and MHCC97-L cells, which may be diminished by cell death inhibitor 3-methyladenine through beclin-1 activation and mammalian target of rapamycin (mTOR) signaling pathway inhibition [90]. In addition, berberine also modifies LC3, an autophagic marker, in human lung cancer A549 cells, indicating that autophagy may play a crucial role in berberine-induced cancer cell death [91].

Berberine also inhibits tumor metastasis and invasion. For example, berberine inhibits 12-O-Tetradecanoylphorbol 13-acetate (TPA)-induced cell migration and blocks prostaglandin E (EP) receptor 4 agonist-induced migration by reducing EP receptors 2 and 4 in A375 and Hs294 cells [92]. Even at low doses, berberine suppresses Rho GTPase activation and induces migration and motility inhibition in HONE1 cells [93]. Berberine also inhibits Rho kinase-mediated Ezrin phosphorylation at Thr (567) in 5-8F cells, leading to a 51.1% inhibition of tumor metastasis to the lymph nodes *in vivo *[94]. A combination of As_2_O_3 _(5 μM) and berberine (10 μM) inhibit the formation of a cell confluent layer by blocking PKCα and ξ, consistent with reduced levels of myelocytomatosis oncogene (Myc), Jun proto-oncogene, metallothionein 1-MMP and MMP-2 [95].

Berberine enhances chemo- and radio-sensitivity, implying its potential as an adjuvant in cancer therapy. Combined with chemotherapy drugs such as cisplatin or As_2_O_3_, berberine exhibits significant cytotoxicity in HeLa and SH-SY5Y cells compared with monotherapy [96,97]. When combined with γ radiation, the apoptotic effect is significantly enhanced in HepG2 cells [98]. Berberine also alleviates chemo-resistance by down-regulating overexpressed transformed mouse 3T3 cell double minute-2 and activating p53 in acute lymphoblastic leukemia cells [99]. Berberine's poor bioavailability makes it less likely to be an independent anti-tumor agent [100-102]. Berberine is nevertheless a potential natural compound for alternative cancer therapy.

### Artemisinin and its derivatives (ARTs)

Artemisinin (Figure 1F) is an active terpene of the Chinese medicinal herb *Artemisia annua *L. (*Huanghuahao*) used in China to treat malaria and fever. ARTs, such as dihydroartemisinin (DHA) and artesunate (Figure 1G), exhibit anti-cancer activities *in vitro *and *in vivo *[103-106]. DHA is one of the main metabolites of ARTs and artesunate is a semi-synthesized derivative of ARTs; both compounds exhibit anti-cancer potentials.

The anti-cancer potential of ARTs has been demonstrated in various cancer cells including those of leukemia and other cancer cells of breast, ovary, liver, lung, pancreas and colon [104,105]. The selective anti-cancer potential of ARTs was related with the expression of different molecules such as c-MYC, cdc25A, EGFR, γ-glutamycysteine synthetase (GLCLR) [105,106]. ARTs also exert anti-cancer effects *in vivo *in multiple cancer types [103,107,108]. For example, either DHA or artesunate has anti-cancer activity against pancreatic cancer xenografts [107,109].

The anti-cancer mechanism of ARTs is likely to be related to the cleavage of the iron- or heme-mediated peroxide bridge, followed by the generation of reactive oxygen species (ROS) [110-112]. According to Efferth *et al. *[113], CCRF-CEM and U373 cells are sensitive to a combined treatment of ARTs and iron (II)-glycine sulfate or holotransferring. Pretreatment with deferoxamine mesylate salt (an iron chelator) visibly reduces DHA-induced apoptosis in HL-60 leukemia cells [104]. The anti-cancer potential of ARTs is possibly connected to the expression of TfR. The synergism of artesunate and iron (II)-glycine sulfate co-treatment is unsuitable for all types of tumor cells [114]. Endoplasmic reticulum stress is partially involved in some cases of ARTs-mediated anti-proliferation [115,116].

ARTs induce cell cycle arrest in various cell types [103,115,117]. For example, DHA and artesunate effectively mediate G1 phase arrest in HepG2 and Hep3B cells [103]. DHA reduces cell number in the S phase in HCT116 colon cancer cells [115]. Interestingly, DHA also arrests the G2 phase in OVCA-420 ovarian cancer cells [117]. Thus, ART-mediated cell cycle arrest is possibly cell type dependent. ARTs also induce apoptotic cell death in a number of cell types, in which the mitochondrial-mediated apoptotic pathway plays a decisive role [104,106]. For instance, DHA enhances Bax and reduces Bcl-2 expression in cancer cells [103,107]. DHA-induced apoptosis is abrogated by the loss of Bak and is largely reduced in cells with siRNA-mediated downregulation of Bak or NOXA [118]. However, DHA activates caspase-8, which is related to the death receptor-mediated apoptotic pathway in HL-60 cells [104]. DHA enhances Fas expression and activates caspase-8 in ovarian cancer cells [119]. DHA also enhances death receptor 5 and activates both mitochondrial- and death receptor-mediated apoptotic pathways in prostate cancer cells [120]. ARTs-induced apoptosis in cancer cells may involve p38 MAPK rather than p53 [103,104].

ARTs inhibit angiogenesis which is a vital process in metastasis [121-124]. DHA inhibits chorioallantoic membrane angiogenesis at low concentrations and decreases the levels of two major VEGF receptors on HUVEC [122]. Conditioned media from K562 cells pre-treated with DHA inhibits VEGF expression and secretion in chronic myeloid leukemia K562 cells, leading to angiogenetic activity decrease [121,124]. Artemisinin inhibits cell migration and concomitantly decreases the expression of MMP2 and the αvβ3 integrins in human melanoma cells [125]. ARTs also regulate the levels of u-PA, MMP2, MMP7 and MMP9 all of which are related to metastasis [126].

ARTs exert synergistic effects with other compounds. Combination of DHA and caboplatin significantly reduces the development of ovarian cancer as compared with DHA only [119]. Combined use of DHA or artesunate with gencitabine inhibits the growth of HepG2 and Hep3B transplanted tumors [103]. Supra-additive inhibition of cell growth in some glioblastoma multiforme cells is observable when artesunate is in combined use with EGFR inhibitor OSI-774 [127]. DHA not only up-regulates death receptor 5 expression but also cooperates with TNF-related apoptosis-inducing ligand (TRAIL) to induce apoptosis in human prostate cancer cells [120]. Therefore, either used alone or in combination with other compounds, ARTs are promising compounds for chemotherapy.

### β-elemene

Elemene (Figure 1H) is a sesquiterpene mixture isolated from more than 50 Chinese herbs and plants, such as *Curcuma wenyujin *Y. H. Chen *et *C. Ling (*Wenyujin*) [128]. Elemene is mainly composed of β- and δ- and γ-elemene, with β-elemene accounting for 60%-72% of all three isoforms. β-elemene exerts anti-cancer potential in brain, laryngeal, lung, breast, prostate, cervical, colon and ovarian carcinomas [128-130]. Elemene shows synergistic effects in combination with other chemotherapeutic drugs [131], leading to the blockade of cell cycle progression by modulating the G2 cell cycle checkpoint and inducing G2/M arrest in human non-small cell lung cancer (NSCLC) and ovarian carcinoma cells while inducing G0/G1 phase arrest in glioblastoma cell lines through phosphorylation of p38 MAPK [129,130,132]. In NSCLC cells, β-elemene induces cell arrest at the G2/M phase by increasing phospho-Cdc2 (Tyr15) and p27/Kip1, and by decreasing phospho-Cdc2 (Thr161) and cyclin B1. Moreover, elemene reduces the expression of Cdc25C, activates Cdc2 and increases Chk2 [129]. β-elemene combined with cisplatin also mediate G2/M cell cycle arrest in chemo-resistant ovarian carcinoma cells through down-regulation of cyclin B1 and Cdc2 by elevating the levels of phosphorylation of Cdc2, Cdc25C, p53, p21/Waf1, p27/Kip1 and GADD45 [130]. β-elemene also induces mitochondrial-mediated apoptosis in prostate cancer and NSCLC cells [128,129]. Combining β-elemene with cisplatin, docetaxel and taxanes significantly increases its inhibitory effect in androgen-independent prostate carcinoma DU145 and PC-3 cells, as well as in NSCLC H460 and A549 cells [131]. β-elemene enhances cellular uptake of taxanes due to the alteration of cell membrane permeability may partly account for its synergistic effects with taxanes [131]. Elemene inhibits the growth of human epidermoid and thyroid cancer cells *in vivo *[133], and passes through the blood-brain barrier [134], suggesting its potential for treating cerebral malignancy.

β-elemene has been approved by China's State Food and Drug Administration as a second class innovative drug and is prescribed as an adjuvant drug for some tumor therapies in China.

### Oridonin

Oridonin (Figure 1I) is a diterpenoid isolated from *Rabdosia rubescens *(Hemsl.) Hara (*Donglingcao*), with its dry raw herb consisting of up to 0.35% of oridonin [135]. *Rabdosia rubescens *(Hemsl.) Hara has long been used to treat sore throat, tonsillitis, and esophageal cancer by native residents of Henan Province. Oridonin was included in the Chinese Pharmacopoeia in 1977. Main chemical constituents of *Rabdosia rubescens *(Hemsl.) Hara are ent-Kaurene diterpenoids, which have multiple biological activities, such as anti-inflammatory, anti-bacterial and anti-tumor effects.

Oridonin significantly inhibits tumor cell proliferation, induces cell cycle arrest and promotes cell death. In anti-proliferation tests, different cell lines exhibited similar sensitivity to oridonin with an IC_50 _of about 40-80 μM after 24 hours of treatment [136-141]. Oridonin induces G2/M cell cycle arrest by up-regulation of heat shock 70 kDa protein 1, serine-threonine kinase receptor-associated protein, translationally controlled tumor protein, stress-induced phosphoprotein 1, trifunctional purine biosynthetic protein adenosine-3 and inorganic pyrophosphatase as well as down-regulation of poly(rC)-binding protein 1 [142] in a p53-independent and p21/Waf1-dependent manner [143]. Induction of apoptosis contributes to oridonin-induced cell death, mainly through mitochondrial-mediated pathways. The up-regulation of Fas, Fas ligand (FasL) and Fas (TNFRSF6)-associated *via *death domain (FADD) expression, as well as the down-regulation of pro-caspase-8 expression suggests that the activation of the Fas/FasL pathway may also be partially involved in oridonin-induced apoptosis [144]. Possible downstream responses include the induction of loss of mitochondrial transmembrane potential [145], the activation of several caspases [136,146], the down-regulation of Bcl-2, the up-regulation of Bax and Bid [136,147] as well as the promotion of cytochrome *c *release [147] and PARP cleavage [148]. However, the regulation of Bcl-xL and participation of caspase-3/9 remain controversial [136,143,146,148-150]. Oridonin-induced intracellular ROS formation may be an initiator of this process [143,151]. Other proteins may also be involved in oridonin-induced cell cycle arrest and apoptosis; these proteins include ERK [144,152], p38MAPK [149], insulin-like growth factor 1 receptor [153], EGFR [154], NF-κB [155], as well as p16, p21/Waf1, p27/Kip1 and c-MYC [156]. Oridonin induce cell death by affecting the balance of apoptosis and necrosis. In A375-S2 cells, low concentrations (34.3 μM) of oridonin induce p53 and ERK-dependent apoptosis whereas high concentrations (137.4 μM) induce necrosis [146]. In L929 cells, oridonin induces a caspase-independent and mitochondria- or MAPK-dependent cell death through both apoptosis and necrosis [139,149]. Similar results are also observed in A431 cells [154]. Oridonin also induces simultaneous autophagy and apoptosis in MCF-7 [157] and HeLa cells [138]. This autophagy may be attributed to the inactivation of Ras, changes in mitochondrial membrane potential [158], activation of PKC, Raf-1 or c-jun N-terminal kinase (JNK) signaling [141] and even NF-κB signaling pathways [159]. Inhibition of autophagy is attributed to apoptotic up-regulation because oridonin-induced apoptosis augmentation is accompanied by reduced autophagy [138] whereas oridonin-induced autophagy inhibits ROS-mediated apoptosis by activating the p38 MAPK-NF-κB survival pathways in L929 cells [160]. Oridonin inhibits DNA, RNA, and protein syntheses [161], decrease telomerase, as well as down-regulate human telomerase reverse transcriptase mRNA expression [162]. The *in vivo *anti-tumor activities of oridonin have been demonstrated in different tumors such as Ehrlich ascites carcinoma, sarcoma-180 solid tumors and in leukemic mice models [163,164].

### Triptolide

Triptolide (Figure 1J) is a diterpenoid triepoxide and the principal active ingredient of *Tripterygium wilfordii Hook. f*. (*Leigongteng*) used in Chinese medicine to treat inflammation and autoimmune diseases [165]. Triptolide exhibits potent anti-inflammation, immunomodulation and anti-tumor activities [166-170]. Triptolide exerts multiple effects on apoptosis, angiogenesis, metastasis and drug-resistance [166-170].

Triptolide is active in pro-apoptosis in diverse tumor cell types including ovarian cancer [166], myeloma [167], myeloid leukemia [168], thyroid carcinoma [169] and pancreatic tumor cells [170]. Many *in vitro *and *in vivo *studies have tried to elucidate the potential mechanism of triptolide; however, conclusions have been inconsistent. Triptolide seems to induce apoptosis *via *different pathways in various cell lines. For example, triptolide induces apoptosis by the overexpression of cytomembrane death receptor in a caspase-8-dependent manner in pancreatic tumor [170] and cholangiocarcinoma cells [171]. Triptolide also promotes apoptosis in leukemic and hepatocarcinoma cells by the mitochondrial-mediated pathway [172,173].

Triptolide is a potent inhibitor of tumor angiogenesis in a zebrafish embryo model and demonstrates potent activities against vessel formation by nearly 50% at 1.2 μM [165]. In a xenograft model, triptolide (0.75 mg/kg/day) blocks tumor angiogenesis and progression in a murine tumorigenesis assay possibly correlated with the down-regulation of proangiogenic Tie2 and VEGFR-2 expression [174]. *In vitro *studies have shown that triptolide inhibits the proliferation of HUVEC. A chick embryo chorioallantoic membrane test shows that triptolide inhibits angiogenesis as well. Triptolide impairs VEGF expression in thyroid carcinoma TA-K cells and down-regulates NF-κB pathway activity; the target genes of triptolide are associated with endothelial cell mobilization in HUVEC [165]. The down-regulation of NF-κB signaling [175], in combination with the inhibition of VEGF expression [176], may be the anti-angiogenesis action of triptolide.

Furthermore, triptolide inhibits tumor metastasis, reducing basal and stimulated colon cancer cell migration through collagen by 65% to 80% and decreasing the expression of VEGF and COX-2 [174]. Triptolide inhibits the expression of multiple cytokine receptors potentially involved in cell migration and cancer metastasis, including the thrombin receptor, CXCR4, TNF receptors and TGF-β receptors [174]. Triptolide also inhibits interferon-γ-induced programmed death-1-ligand 1 surface expression whose up-regulation is an important mechanism of tumor immune evasion in human breast cancer cells [177]. Triptolide inhibits the experimental metastasis of melanoma cells to the lungs and spleens of mice [178]. Moreover, triptolide inhibits the migration of lymphoma cells *via *lymph nodes, a result which may be related to its anti-proliferative effects and blockage of the SDF-1/CXCR4 axis [179].

Triptolide enhances the anti-neoplastic activity of chemotherapy [180,181]. The combination index-isobologram indicates that the effect of triptolide on 5-FU is synergistic on colon carcinoma [180]. In a tumor xenograft model, the combined effects of triptolide (0.25 mg/kg/day) and 5-FU (12 mg/kg/day) on the growth of colon carcinoma are superior to those of individual agents [180]. Triptolide is synergistic with other anti-cancer agents or therapies including hydroxycamptothecin [181], idarubicin, AraC [182], TRAIL [183] and ionizing radiation [184]. These results indicate the therapeutic potential of triptolide in treating cancer.

### Ursolic acid (UA)

UA (Figure 1K) is a ubiquitous pentacyclic triterpenoid compound from many plants such as *Ligustrum lucidum *Ait. (*Nuzhen*). UA exerts proliferation inhibition in human ovarian cancer CAOV3 cells and doxorubicin-resistant human hepatoma R-HepG2 cells [185,186]. UA disrupts cell cycle progression and induces necrosis in a clonal MMTV-Wnt-1 mammary tumor cell line [187]. Eight novel UA derivatives with substitutions at positions C-3, C-11, and C-28 of UA show cytotoxicity to some degree in HeLa, SKOV3 and BGC-823 *in vitro; *only one derivative exhibits more potent cytotoxicity than UA [188].

UA induces apoptosis *via *both extrinsic and intrinsic signaling pathways in cancer cells [189]. In PC-3 cells, UA inhibits proliferation by activating caspase-9 and JNK as well as FasL activation and Akt inhibition [190]. A significant proliferation inhibition and invasion suppression in both a dose- and time-dependent manner is observed in highly metastatic breast cancer MDA-MB-231 cells; this inhibition is related to the down-regulation of MMP2 and u-PA expression [191]. Moreover, UA reduces IL-1β- or TNF-α-induced rat C6 glioma cell invasion and inhibits the interaction of ZIP/p62 and PKC-ζ [192]. Nontoxic UA concentrations inhibit vessel growth in rat aortic ring and down-regulate matrix MMPs such as MMP2 and MMP9 [193]. In other cancer cell lines, such as Hep3B, Huh7 and HA22T cells, UA exerts a potential anti-angiogenic effect by decreasing HIF-1α, VEGF and IL-8 gene expression [194].

### Shikonin

Shikonin (Figure 1L) is a natural anthraquinone derivative isolated from the roots of *Lithospermum erythrorhizon *(*Zicao*) and exerts anti-tumor effects mainly by inhibiting cell growth and inducing apoptosis. The underlying molecular mechanisms vary with cell types and treatment methods. Shikonin induces apoptosis in a classic caspase-dependent pathway in cervical, bladder and melanoma cancer cells [195-198]. Shikonin induces necroptosis regardless of the drug concentration in caspase-3-negative MCF-7 cells [199]. Different concentrations of shikonin induce either apoptosis or necroptosis, and necroptosis converts to apoptosis in the presence of Nec-1 in HL-60 and K562 cells [200]. The growth inhibition and apoptosis induced by shikonin in some cancer cells may be attributed to the inactivation of NF-κB activity or increasing Annexin V signal and CD95 (Fas/APO) expression [201,202]. Shikonin also induces apoptosis *via *ROS production in osteosarcoma and Bcr/Abl-positive CML cells [203,204].

Several different mechanisms contribute to the anti-cancer activities of shikonin. For example, shikonin suppresses proteasomal activities thereby inhibiting tumor growth in both H22 allografts and PC-3 xenografts [205]. Shikonin also inhibits topoisomerase II [206] and down-regulates ER2 and activates NFE2-related factor 2 as an anti-estrogen agent in human breast cancer [207,208]. Shikonin modulates an estrogen enzyme by down-regulating the expression of steroid sulfatase which is important for estrogen biosynthesis [205]. Shikonin inhibits tumor invasion *via *the NF-κB signaling pathway in human high-metastatic adenoid cystic carcinoma cells [209]. Therefore, shikonin may directly or indirectly inhibit or modulate disease-related cellular targets in cancer.

### Emodin

Emodin (Figure 1M) is a natural anthraquinone derivative isolated from *Rheum palmatum *L. (*Zhangyedahuang*), with its dry raw herb consisting of up to 0.20 mg/100 mg of emodin [210]. Emodin exerts anti-tumor activity against various human cancers [211]. Emodin induces cell cycle arrest and apoptosis in cancer cells [212-214] and the oxidative injury acts upstream of anti-proliferation. Emodin inhibits IL-6-induced Janus-activated kinase 2/STAT3 pathways and induces apoptosis in myeloma cells *via *the down-regulation of Mcl-1 [213]. Emodin down-regulates androgen receptors and inhibits prostate cancer cell growth [215]. Moreover, emodin stabilizes topoisomerase II-DNA cleavage complexes, thereby inducing DNA double-strand breaks [216]. The suppression of excision repair cross complementation 1 (ERCC1) and Rad51 expression through ERK1/2 inactivation is vital in emodin-induced cytotoxicity in human NSCLC cells [217].

Emodin inhibits basic fibroblast growth factor (bFGF)-induced proliferation and migration in HUVEC and VEGF-A-induced tube formation [218]. Emodin inhibits tumor cell migration through suppression of the phosphatidylinositol 3-kinase-Cdc42/Rac1 pathway [219]. The disruption of the membrane lipid raft-associated integrin signaling pathway by emodin may inhibit cell adhesion and spreading [220].

Emodin sensitizes chemotherapy associated with ROS production [221,222]. In combined use with cisplatin, emodin elevates ROS generation and enhances chemosensitivity in DU-145 cells, accompanied by the down-regulation of MDR1 expression and suppression of HIF-1α transactivation [223]. Emodin enhances the sensitivity of gallbladder cancer SGC996 cells to platinum drugs *via *glutathione depletion and multidrug resistance-related protein 1 down-regulation [224]. The mechanisms of the synergistic effects of emodin with cisplatin or gencitabin may be attributed to the emodin-induced down-regulation of ERCC1 and Rad51 expression, respectively [225,226]. These results suggest that emodin may be used as an adjuvant to enhance the anti-cancer effects of chemotherapeutic agents.

### Ginsenoside Rg_3_

Extracted from *Panax ginseng *C.A. Mey. (*Renshen*) and *Panax quinquefolius *L., Araliaceae (*Xiyangshen*), ginsenoside Rg_3 _(Figure 1N) is a biologically active component with both *in vitro *and *in vivo *anti-cancer activities [227,228]. The anti-proliferative mechanism of ginsenoside Rg_3 _is associated with the inactivation of NF-κB [229,230], modulation of MAPKs [231] and the down-regulation of Wnt/β-catenin signaling [232]. Ginsenoside Rg_3 _affects the ephrin receptor pathway in HCT-116 human colorectal cancer cells [233]. The anti-proliferative mechanism of ginsenoside Rg_3 _is also associated with the molecules of mitotic inhibition, DNA replication, repair, and growth factor signaling [234].

Ginsenoside Rg_3 _inhibits the proliferation of HUVEC and suppresses the capillary tube formation of HUVEC on a matrigel at nanomole scales in the presence or absence of VEGF. Ginsenoside Rg_3 _attenuates VEGF-induced chemo-invasion of HUVEC and *ex vivo *microvascular sprouting in rat aortic ring. bFGF-induced angiogenesis may be abolished by ginsenoside Rg_3 _[227]. In lung metastasis models of ovarian cancer, ginsenoside Rg_3 _decreases the number of tumor colonies in the lung and vessels oriented toward the tumor mass [235]. This effect may be partially due to the inhibition of angiogenesis and the decrease in MMP9 expression [235].

Ginsenoside Rg_3 _increases the efficacy of cancer chemotherapy. Combined treatments with ginsenoside Rg_3 _enhance the susceptibility of colon cancer cells to docetaxel, paclitaxel, cisplatin and doxorubicin; the mechanism of such an enhancement is related to the inhibition of the constitutively activated NF-κB [229]. A similar phenomenon has been observed in prostate cancer cells, in which the combination of ginsenoside Rg_3 _and docetaxel more effectively induces apoptosis and G1 cell cycle arrest, accompanied by the inhibition of NF-κB activity [230]. Low-dose administration of cyclophosphamide (CTX) with ginsenoside Rg_3 _increases the efficacy of targeting the tumor microvasculature and the two-drug combination treatment results demonstrate the longest patient survival rates [236]. Ginsenoside Rg_3 _combined with gemcitabine not only enhances the efficacy of tumor growth suppression and survival prolongation, but also decreases VEGF expression and microvessel density in tumors [228].

## Conclusion

Natural products such as GA, curcumin, β-elemene *et al. *derived from Chinese medicinal herbs are potential candidates for anti-cancer therapeutic drugs.

## Abbreviations

5-FU: 5-fluorouracil; AIF: apoptosis inducing factor; AP-1: activator protein-1; ARTs: artemisinin and its derivatives; ATRA: all-trans retinoic acid; bFGF: basic fibroblast growth factor; CDKs: cyclin-dependent kinases; CTX: cyclophosphamide DHA: dihydroartemisinin; DPD: dihydropyrimidine dehydrogenase; EGFR: epidermal growth factor receptor; ERK1/2: extracellular signal-regulated kinase 1/2; FasL: Fas ligand; GA: gambogic acid; HDMEC: human dermal microvascular endothelial cells; HUVEC: human umbilical vascular endothelial cells; ICAM-1: intercellular cell adhesion molecule-1; IL-1β: interleukin-1β; LC3: light chain 3; MMP2: matrix metalloproteinase-2; MMP9: matrix metalloproteinase9; MRP1: multidrug resistance-associated protein 1; NF-κB: nuclear factor-kappa B; P-gp: P-glycoprotein; PI3K: phosphoinositide 3-kinase; ROS: reactive oxygen species; STAT: signal transducer and activator of transcription; STS: steroid sulfatase; TfR: transferrin receptor; TPA: 12-O-tetradeca noylphorbol-13-acetate; TRAMP: transgenic adenocarcinoma of the mouse prostate; UA: ursolic acid; u-PA: urokinase plasminogen activators; VCAM-1: vascular cell adhesion molecule-1; VEGF: vascular endothelial growth factor; VEGFR1:vascular endothelial growth factor receptor 1; VEGFR2: vascular endothelial growth factor receptor 2.

## Competing interests

The authors declare that they have no competing interests.

## Authors' contributions

WT, JJL, MQH, YBL, MWC, GSW, JG, ZFZ, ZTX, YYD and XPC wrote the manuscript (WT wrote berberine; JJL wrote GA and ARTs; MQH wrote emodin and ginsenoside Rg3; YBL wrote cucurmin; MWC wrote silibinin; GSW wrote shikonin; JG wrote wogonin; ZFZ wrote β-elemene; ZTX wrote triptolide; YYD wrote UA; XPC wrote oridonin). JJG drew the chemical structures in Figure 1. WT, JJL and XPC revised the manuscript. YTW designed and supervised this work. All authors read and approved the final version of the manuscript.
